# Microbiome Compositions and Resistome Levels after Antibiotic Treatment of Critically Ill Patients: An Observational Cohort Study

**DOI:** 10.3390/microorganisms9122542

**Published:** 2021-12-09

**Authors:** Karen Leth Nielsen, Markus Harboe Olsen, Albert Pallejá, Søren Røddik Ebdrup, Nikolaj Sørensen, Oksana Lukjancenko, Rasmus L. Marvig, Kirsten Møller, Niels Frimodt-Møller, Frederik Boëtius Hertz

**Affiliations:** 1 Department of Clinical Microbiology, Copenhagen University Hospital—Rigshospitalet, Blegdamsvej 9, 2100 Copenhagen, Denmark; niels.frimodt-moeller@regionh.dk (N.F.-M.); frederik.boetius.hertz@regionh.dk (F.B.H.); 2 Department of Neuroanaesthesiology, The Neuroscience Centre, Copenhagen University Hospital—Rigshospitalet, Blegdamsvej 9, 2100 Copenhagen, Denmark; markus.harboe.olsen@regionh.dk (M.H.O.); Soeren.Roeddik.Ebdrup@regionh.dk (S.R.E.); Kirsten.Moeller.01@regionh.dk (K.M.); 3 Clinical Microbiomics, Fruebjergvej 3, 2100 Copenhagen, Denmark; apalleja@clinical-microbiomics.com (A.P.); Nikolaj@clinical-microbiomics.com (N.S.); Oksana@clinical-microbiomics.com (O.L.); 4 Department of Genomic Medicine, Copenhagen University Hospital—Rigshospitalet, Blegdamsvej 9, 2100 Copenhagen, Denmark; rasmus.lykke.marvig@regionh.dk; 5 Department of Clinical Medicine, Faculty of Health and Medical Sciences, University of Copenhagen, Blegdamsvej 3B, 2200 Copenhagen, Denmark

**Keywords:** metagenomics, microbiome, microbiota, antimicrobial resistance, antimicrobial treatment

## Abstract

Hospitalization and treatment with antibiotics increase the risk of acquiring multidrug-resistant bacteria due to antibiotic-mediated changes in patient microbiota. This study aimed to investigate how broad- and narrow-spectrum antibiotics affect the gut microbiome and the resistome in antibiotic naïve patients during neurointensive care. Patients admitted to the neurointensive care unit were treated with broad-spectrum (meropenem or piperacillin/tazobactam) or narrow-spectrum antibiotic treatment (including ciprofloxacin, cefuroxime, vancomycin and dicloxacillin) according to clinical indications. A rectal swab was collected from each patient before and after 5–7 days of antibiotic therapy (*N* = 34), respectively. Shotgun metagenomic sequencing was performed and the composition of metagenomic species (MGS) was determined. The resistome was characterized with CARD RGI software and the CARD database. As a measure for selection pressure in the patient, we used the sum of the number of days with each antibiotic (antibiotic days). We observed a significant increase in richness and a tendency for an increase in the Shannon index after narrow-spectrum treatment. For broad-spectrum treatment the effect was more diverse, with some patients increasing and some decreasing in richness and Shannon index. This was studied further by comparison of patients who had gained or lost >10 MGS, respectively. Selection pressure was significantly higher in patients with decreased richness and a decreased Shannon index who received the broad treatment. A decrease in MGS richness was significantly correlated to the number of drugs administered and the selection pressure in the patient. Bray–Curtis dissimilarities were significant between the pre- and post-treatment of samples in the narrow group, indicating that the longer the narrow-spectrum treatment, the higher the differences between the pre- and the post-treatment microbial composition. We did not find significant differences between pre- and post-treatment for both antibiotic spectrum treatments; however, we observed that most of the antibiotic class resistance genes were higher in abundance in post-treatment after broad-spectrum treatment.

## 1. Introduction

The microbiota of the gastro-intestinal (GI) tract of humans consists of thousands of bacterial species [1] and the population is dominated by anaerobic bacteria, with less than 1% being facultative aerobic bacteria. Endogenous microbiota exhibits a defensive barrier against colonization by pathogenic bacteria; a phenomenon known as colonization resistance [1]. Colonization resistance is fundamental in the prevention of colonization and infection of individuals exposed to exogenous multidrug-resistant or pathogenic bacteria [2,3]. The gut microbiota can inhibit pathogenic or opportunistic bacteria directly via competition (e.g., production of bacteriocins, competition for iron, carbon or other nutrients, production of short-chain fatty acids (SCFAs) and modulation of oxygen concentration) or indirectly by regulating the host immune response [4]. In particular, loss of anaerobic bacteria has been correlated with loss of colonization resistance [2,3]. Enterobacterales (e.g., *Escherichia coli* and *Klebsiella pneumoniae*) and *Enterococcus* spp. are generally present in low abundance in the human gut. However, if the gut microbiota is altered, overgrowth of Enterobacterales and *Enterococcus* spp. is usually observed, including overgrowth of ESBL- and carbapenemase-producing Enterobacterales as well as vancomycin-resistant *E. faecium* [2,3,5].

One of the most distinctive changes to the gut community is antimicrobial therapy [5,6]. In patients colonized by multidrug-resistant microorganisms, antibiotics may promote high-density colonization and an increase in the overall gene pool of resistance genes, which subsequently increase the risk or further spread, environmental contamination and infection [7]. Antimicrobial stewardship, interpreted as avoiding unnecessary antimicrobial therapy, shortening the duration of treatment, and favoring antibiotics with a narrow spectrum of activity, is crucial in avoiding the abolishment of the colonization resistance. Use of narrow-spectrum antibiotics aims to avoid general anti-anaerobe activity, avoiding a decrease in microbiota diversity. The effect of antibiotics on the microbiota is evident on all epithelial linings, where susceptible bacteria are replaced with resistant and/or pathogenic bacteria [8]. What replaces the endogenous microbiota depends on the antibiotic administered; thus, broad-spectrum antibiotics will have an effect on many different bacterial clades, and has been shown to cause growth of resistant bacteria and fungi on the epithelial linings after treatment [8]. However, antimicrobial cocktails, including broad-spectrum antimicrobials, drastically reduced the gut microbiota’s alpha diversity in healthy young men in a study by Palleja et al. [9]. Furthermore, the decrease in the usual commensals was followed by an increase in Enterobacterales (such as *Klebsiella* spp. or *E. coli*) [9].

There are only few studies on the effect of antimicrobial consumption on the gut microbiome and resistome in intensive care units. Palleja et al. studied the gut composition and alterations in the resistome of healthy individuals under a 4-day intervention with a cocktail of three broad-spectrum antibiotics commonly administered in ICU [9]. They identified an increase in beta-lactam-resistant species after treatment as well as compositional changes in correlation to in vitro studies [9]. A previous study from McDonald et al. [10] showed strong but varying changes in the microbiome of critically ill patients, including removal of endogenous species.

The purpose of this study was to evaluate the effect of common antibiotics on the intestinal microbiome composition, diversity and resistome using real-world data from neurocritically ill patients. These patients were chosen as they have a higher chance of being antibiotic naive on admission compared to more conventional critically ill patients, while still being at high risk of exposure to antibiotics during their hospital stay. By studying the intestinal microbiota before and after exposure to antibiotics, we aimed to identify patterns in antimicrobial consumption, classified as broad and narrow, associated with changes in the gut diversity and resistome.

## 2. Materials and Methods

### 2.1. Study Design and Settings

This was an observational cohort study, where we analyzed the microbiome of antibiotic naive patients admitted to the neurointensive care unit (NICU) at Rigshospitalet, Copenhagen, Denmark. Rigshospitalet is a highly specialized, tertiary referral hospital with 1300 beds; the NICU admits around 1000 patients annually with traumatic or spontaneous brain injury, intracranial hemorrhage, cervical spinal cord injury and neurological diseases with a need for intensive care.

Between April 2018 and January 2019, we recruited patients admitted to NICU using a convenience-sample approach. Patients were eligible for one baseline rectal swab performed at admission, prior to initiation of antimicrobial treatment, and one study sample (2nd rectal swab) after 5–7 days of antibiotic treatment.

### 2.2. Ethical Concerns

There were no ethical concerns for this study. This was a non-intervention study. All patients were treated according to the standard of care. Relevant healthcare professionals treated all included patients according to current treatment guidelines, independently of this study. Human DNA was not of interest. Any DNA sequencing reads with homology to the human reference genome were discarded and destroyed prior to data analyses. Research ethical approvals were given by The Regional Committee of Danish Data Protection Agency and the Regional Committee of Health Research Ethics Committee (j.nr.: 2012-58-0004 and j-nr.: H-17034766). Written informed consent was provided by the next of kin.

### 2.3. Study Participants and Samples

We enrolled 37 who were administered antibiotics after the baseline sample and 5 patients who did not receive any antibiotics (controls). Two of the controls were also included in the patient group as they received antibiotics after delivering the control samples. In total, 84 samples were sequenced. Eight samples were excluded: One sample failed in sequencing and both samples from this individual were therefore excluded; three samples had mixed metadata, and hence, were excluded from the analyses. One patient was excluded from the study as the patient chart could not rule out that antimicrobial treatment had been administered; one sample was a duplicate of another sample, and thus, were excluded from the further analysis. Hence, a total of 78 samples were considered in the analysis, belonging to 34 cases and 5 controls.

### 2.4. Antibiotic Regimens

We grouped patients according to the antibiotic regimens administered: Treatment with antibiotics of broad spectra (meropenem and piperacillin/tazobactam) and narrow spectra (all other regimens), respectively. Meropenem and piperacillin/tazobactam are active against Gram-positive and Gram-negative bacteria and affect both aerobic and anaerobic bacteria. If the patients received meropenem or piperacillin/tazobactam at any time during the sampling period, they were classified as belonging to the broad-spectrum group. An overview of the treatments administered can be found in Table 1.

### 2.5. Laboratory Analyses

After collection, rectal swabs were refrigerated and shipped to the Department of Clinical Microbiology, Rigshospitalet, for storage. We added 250 µL glycerol to each individual sample that was subsequently frozen at −80 °C until all samples had been collected.

### 2.6. DNA Extraction, Library Preparation and Sequencing

Prior to DNA extraction the rectal swabs were defrosted on ice and centrifuged at 300× *g* for 10 min, to pellet a large part of the epithelial cells. The supernatant was carefully transferred to a new Eppendorf. DNA was extracted using NucleoSpin^®^ 96 Soil (Macherey-Nagel). Bead beating was done on a Vortex-Genie 2 horizontally for 5 min. The genomic DNA was randomly sheared into fragments of around 350 bp using ultrasonic interruption (Bioruptor Pico, Diagenode SA). The fragmented DNA was applied for library construction using NEBNext Ultra II Library Prep Kit for Illumina (New England Biolabs, Ipswich, MA, USA). The prepared DNA libraries were evaluated using a Qubit 2.0 fluorometer and Agilent 2100 Bioanalyzer. Quantitative real-time PCR (qPCR) was used to determine the concentration of the final library before sequencing. The library was sequenced using 2 × 150 bp paired-end sequencing on an Illumina platform (Novaseq6000).

### 2.7. Sequencing Data Quality Control

Quality control of the raw FASTQ files was performed using KneadData v. 0.6.1. Human reads were removed with Trimmomatic v. 0.36; the reads were quality trimmed by removing the Nextera adapter sequences, leading and trailing bases with a Phred score below 20, and trailing bases in which the Phred score over a window of size 4 drops below 20. Trimmed reads shorter than 100 bases were discarded. Reads that mapped to the human reference genome GRCh38 (Bowtie2 v. 0.2.3.2 using default settings) were discarded. Only read pairs in which both reads passed filtering were retained; these were classified as high-quality non-host (HQNH) reads.

### 2.8. Mapping Reads to Microbial Gene Catalog

As a reference gene catalog, we used the Clinical Microbiomics Human Gut 22M non-redundant gene catalog (in total 22,459,186 microbial genes), which was created from >5000 deep-sequenced human gut specimens. For metagenomic species (MGS) abundance profiling, we used the Clinical Microbiomics HGMGS v.2.3 containing 1273 MGS, which have highly coherent abundance and base composition in a set of 1776 independent reference human gut samples [11]. HQNH reads were mapped to the gene catalog using BWA mem v. 0.7.16a with options to increase accuracy (-r 1 -D 0.3). PCR/optical duplicates were removed using SAMtools v. 1.6. An individual read was considered mapped to a gene if the mapping quality (MAPQ) was ≥20 and the read aligned with ≥95% identity over ≥100 bp. However, if a read failed to align to the gene sequence with >10 bases at either end, it was considered unmapped. Reads meeting the alignment length and identity criteria but not the MAPQ threshold were considered multi-mapped. Reads failing the alignment length or identity criteria were considered unmapped. Each read pair was counted as either (1) mapped to a specific gene, if one or both individual reads mapped to a gene, or (2) multi-mapped, if neither read was mapped, and at least one was multi-mapped, or (3) unmapped, if neither individual reads mapped. If the two reads each mapped to a different gene, the gene mapped by read 1 was counted but not the gene mapped by read 2. A gene count table was created with the number of mapped read pairs for each gene (Figure S1). We obtained an average of 40.9 M read pairs per sample with a minimum of 28.7 M read pairs. The sequence quality was very high on most of the samples (Figure S1), but some samples exhibited a high percentage of host DNA (on average we observed 7.5 M host reads). On average, 22.2 M read pairs per sample could be mapped to the microbial gene catalog, representing on average 83.8% of the high-quality non-host (HQNH) reads (min = 31.7%, Table 1).

### 2.9. MGS Relative Abundance Calculation

For each MGS, a signature gene set was defined as the 100 genes optimized for accurate abundance profiling of the MGS. An MGS count table was created by counting the number of reads mapped to the MGS signature genes per sample. An MGS was considered detected if reads from a sample mapped to at least three of its signature genes; measurements that did not satisfy this criterion were set to zero. Based on internal benchmarks, this threshold results in 99.6% specificity. The MGS count table was normalized according to the effective gene length and then normalized sample-wise to sum to 100%, resulting in relative abundance estimates for each MGS. Downsampled (rarefied) MGS abundance profiles were calculated by random sampling without replacement from each sample in the MGS counts table. Values with fewer than three counts after downsampling were set to zero, and the counts table was normalized according to effective gene length and then normalized to sum to 100%.

### 2.10. Resistome Profiling

To annotate the 22 M human microbial gene catalog to the antibiotic-resistance genes (ARG) we used the Comprehensive Antibiotic Resistance Database (CARD) [12]. Catalog genes were assigned to a CARD model by using the CARD RGI software v. 4.2.0 and the CARD database v. 3.0.0 and requiring a hit scoring above the ARG family specific threshold. The top hit was taken if several were achieved. Among the four canonical ARG models available in CARD, we only used the protein homolog models. When mapping the protein homolog models, we assume that genes highly similar to an ARG model (>95% nt similarity) will confer this functional capacity.

### 2.11. Diversity Analyses

For microbiotas, dysbiosis is characterized by a decline in alpha diversity and loss of colonization resistance. Alpha diversity describes the variation of microbes in a single sample while beta diversity describes the variation in microbial communities between samples. Alpha diversity was calculated as richness (number of metagenomic species observed in a sample) and as the Shannon index, which, in addition, accounts for the abundance evenness of the species (i.e., a large Shannon index value is given by the presence of many species with well-balanced abundances). Beta-diversity was calculated using Bray–Curtis dissimilarity among samples, which explains differences in microbial abundances between two samples based on the MGS relative abundances; it is given as values from 0 to 1, where 0 means that both samples share the same species at exactly the same abundances, as opposed to values of 1, indicating that samples do not share any species. To visualize the sample dissimilarities, Bray–Curtis dissimilarities among samples were projected onto the first two dimensions of a principal coordinate analysis (PCoA). Each dot represents a sample. The further the dots are separated, the larger the dissimilarity between their microbial compositions. Both alpha and beta diversity were calculated from the downsized MGS abundances.

### 2.12. Statistics

To identify which MGSs changed differently for both treatments over time we used linear mixed-effects models (LMM), regressing the log10-transformed abundance of taxa or ARG (at antibiotic class) to the interaction antibiotic spectrum (“Broad” and “Narrow”) and timepoints (“Pre-” and “Post-” treatment), and using a random intercept model where patient was considered the random effect. We were interested in identifying MGSs whose antibiotic spectrum and timepoint interaction term was significant, as these are the species upon which there is a differential effect of treatment using different spectrum antibiotics. The *p*-values for each model term was assessed by comparing with the log likelihood-ratio test of the models including and not including the term. Statistical comparisons between pre- and post-treatment were performed using Wilcoxon signed-rank tests, while between the antibiotic-spectrum groups were performed using two-sided Mann–Whitney U (MWU) tests. Where multiple hypotheses were evaluated in parallel, the Benjamini–Hochberg method was used to control for the false discovery rate, considering all hits below an FDR of 10% as significant. Using pre- versus post-treatment test *p*-values and fold changes, we performed post-test Taxon Set Enrichment Analysis (TSEA) to identify whether predefined groups of taxa were statistically overrepresented within pre- or post-treatment for both treatments.

## 3. Results

### 3.1. Study Group Characterization

This was a non-intervention study and patients were included from clinical settings. We therefore investigated actual antibiotic treatment regimens at the NICU and not standardized or controlled regimens. Consequently, the study is biased towards the utilization of broad-spectrum antibiotics (broad *n* = 27; narrow *n* = 7), which may affect the power of our analyses. We tested for differences in covariates between broad and narrow spectrum with respect to duration of treatment, number of antibiotic drugs and selection pressure measured as the number of antibiotic days (the sum of days each antibiotic was administered) (Figure S2). Treatment groups differed with respect to antibiotic days (MWU test; *p* = 0.034) and number of drugs (MWU test; *p* = 0.049). There was, however, no significant difference between the duration of treatment (MWU test; *p* = 0.22). Fewer antibiotics were administered for a shorter period in the narrow treatment group.

### 3.2. Higher Number of Drugs and Selection Pressure for Decreased MGS Diversity Samples

**Figure 1:**
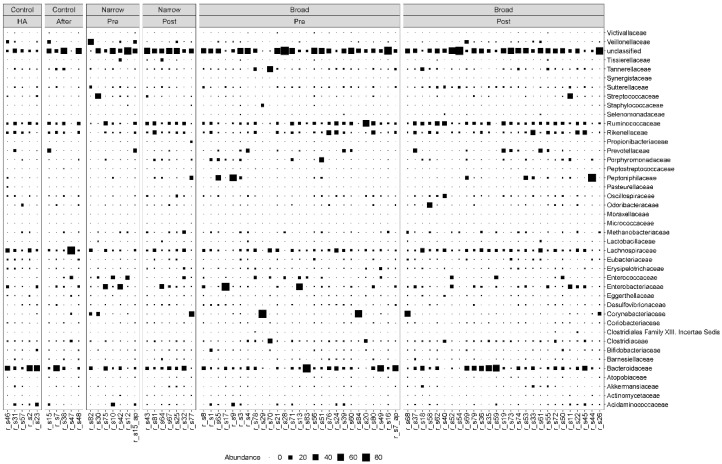
Taxonomic composition at the family level. Bubble plot showing the abundance estimates for the 78 samples included in the analysis. MGS abundances were summarized at the family level and shown separately for each antibiotic spectrum at each timepoint. The relative abundances are in percentages. HA: hospital admission.

We found that the MGS abundance varied among patients regardless of which group they belonged to. Bacteroidetes and Firmicutes were the most dominant phyla, before and after antibiotic treatment as well as in the controls. The most dominant families for both the case and control samples were Bacteroidaceae, Lachnospiraceae, Ruminoccoccaceae and Rikenellaceae (Figure 1). We also observed more modest fluctuations of Enterococcaceae, Enterobacteriaceae, Corynebacteriaceae and Clostridiaceae (Figure 1).

**Figure 2:**
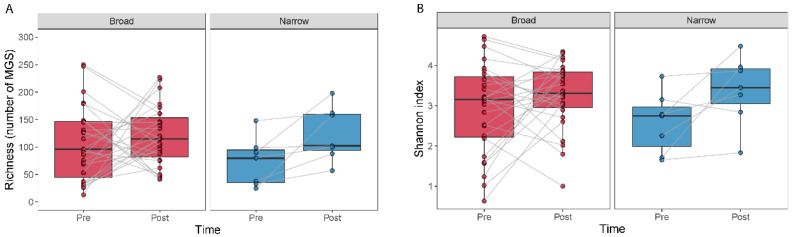
Alpha diversity measured as the number of MGSs detected and by the Shannon index for before and after treatment. Boxplots show the number of MGS detected (**A**) or the Shannon index (**B**) calculated for the samples at each time point. Lines join samples from the same subject.

The effect of antibiotic treatment on the diversity within a sample (alpha diversity) was studied by comparing MGS richness and the Shannon index (Figure 2). We observed an increase in richness (Wilcoxon signed-rank test, *p* = 0.016) and a tendency for an increase in the Shannon index (Wilcoxon signed-rank test, *p* = 0.078) for the narrow-spectrum samples (Figure 2).

**Figure 3:**
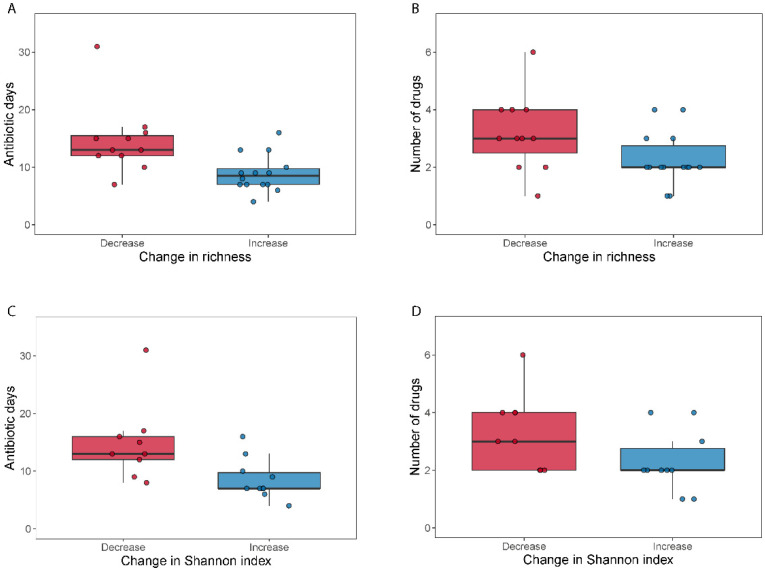
Selection pressure (antibiotic days) and number of antibiotics used related to a change in alpha diversity. Boxplots (**A**–**D**) show the total days on antibiotics and the number of antibiotics for patients who either decreased (“Decrease”) or increased (“Increase”) their diversity (calculated as MGS richness or Shannon index) during broad-spectrum antibiotic treatment.

We did not find differences in alpha diversity, neither in richness (Wilcoxon signed-rank test, *p* = 0.49) nor through the Shannon index (Wilcoxon signed-rank test, *p* = 0.41) within the broad-spectrum group (Figure 2). However, we observed two populations among the broad-spectrum patients; some markedly decreased in diversity and some increased (Figure 2). We therefore studied the possible differences between these two populations that could explain this observation. To stratify the patients based on richness change, we assigned patients that had gained more than ten species to the “Increase” group and those who had lost more than ten species to the “Decrease” group. Patients who had lost or gained less than 10 species were discarded in this analysis. We did the same to stratify patients based on the Shannon index using a threshold of 0.5. The selection pressure (antibiotic days) was significantly higher in patients with a decreased richness (MWU test; *p* = 0.0054) and Shannon index (MWU test; *p* = 0.012) (Figure 3). There was also a tendency among the patients with a decreased richness and Shannon diversity to have had administered a higher number of drugs (MWU tests; for richness *p* = 0.063; for Shannon index *p* = 0.086; Figure 3). Comparing broad and narrow antibiotic treatment we found no significant differences between the two groups regarding MGS richness and Shannon diversity between the antibiotic treatments at pre- (MWU test, *p* > 0.33) or post-treatment (MWU test, *p* > 0.61), indicating no diversity differences between the groups when treatment started and when it ended (Figure 2).

### 3.3. Clostridiales Species Were Enriched in Post-Treatment Samples

**Figure 4:**
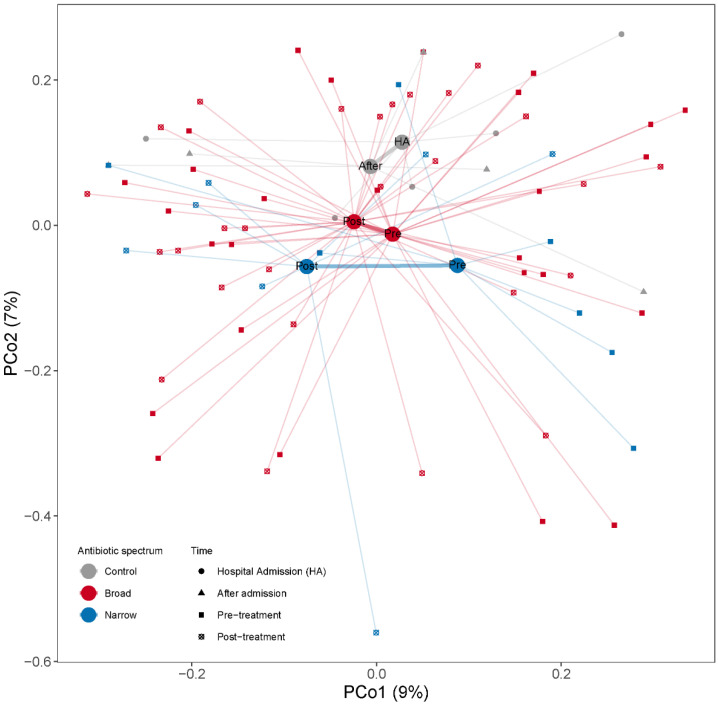
PCoA based on Bray–Curtis dissimilarities among samples calculated from MGS relative abundances to illustrate changes in microbial community composition between the samples. Samples are color coded by the antibiotic treatment (Control, Broad and Narrow spectrum). The mean of all the samples belonging to each antibiotic treatment group is indicated with a larger dot (centroid). All samples belonging to the same antibiotic treatment point are connected by a thin line to their respective centroid. Centroids from the same antibiotic spectrum are connected by a thicker line. The *x*- and *y*-axes show the microbial variance explained by each principal coordinate.

Changes in overall microbial community composition were evaluated by calculating the Bray–Curtis dissimilarities among samples. When we projected the dissimilarities into the two dimensions of a PCoA we observed that the samples were quite dispersed within each group with no clear group separation (Figure 4). To assess the effect of antibiotic spectrum, time and other factors (number of antibiotics, duration of treatment or selection pressure) on the microbiome composition, we used PERMANOVA, assessing the marginal effects and adjusting by subject when more than one sample from one subject is included. When we regressed the dissimilarities among the samples to time (pre- and post-treatment), we did not find any association between time and beta-diversity (PERMANOVA test, *p* = 0.42, R^2^ = 1.6%). We did not find significant compositional differences between narrow and broad-spectrum antibiotics pre- (PERMANOVA test, *p* = 0.54, R^2^ = 2.9%) or post-treatment (PERMANOVA test, *p* = 0.72, R^2^ = 2.7%). We also studied the effect of length of treatment (as antibiotic days) and number of drugs used on the overall microbial composition and did not find any significant compositional differences associated with the treatment modalities. We did not find that the number of antibiotics, duration of treatment or antibiotic days had a significant influence on the post-treatment microbial structure (PERMANOVA tests; *p*_number of antibiotics_ = 0.81, R^2^_number of antibiotics_ = 2.6%; *p*_duration of treatment_ = 0.71, R^2^_duration of treatment_ = 2.7%; *p*_antibiotic days_ = 0.60, R^2^_antibiotic days_ = 2.9%).

We next studied whether there was a correlation between these antibiotic treatment-related parameters and the Bray–Curtis dissimilarities between pre- and post-treatment samples from the same patient (Table 2). Among the narrow-spectrum patients we observed a significant correlation among the Bray–Curtis dissimilarities between the pre- and post-treatment sample of a subject and for the duration of treatment (Spearman correlation, rho = 0.78, *p* = 0.038) and antibiotic days (Spearman correlation, rho = 0.77, *p* = 0.039), indicating that the longer the narrow-spectrum antibiotic treatment, the higher the differences between the pre- and the post-treatment bacterial composition (Table 2).

To identify the MGSs that changed differently due to treatment over the time, we used linear mixed models (LMM), regressing MGS abundances to the interaction of the antibiotic spectrum group (“Broad” and “Narrow”) and time (“Pre” and “Post”), considering subject as a random effect (see methods) and only considering the MGSs present in at least 10 samples (in total 559 MGSs). We were interested in identifying the MGSs whose antibiotic spectrum and time interaction term was significant, as these are the species upon which there is a differential effect of treatments over time. We observed 15 MGS species that changed over time in an antibiotic spectrum-specific manner (Figure S3, LMM, unadjusted *p*_spectrum:time_ interaction < 0.05). Most of these species were *Clostridiales* and *Bacteroides* species together with *Blautia wexlerae* and *Actinomyces oris*, which had similar abundance levels in pre- and post-broad-spectrum treatment, but were mostly enriched in post-treatment for Narrow-spectrum treatment (Figure S3, see statistics and fold changes for the significant MGSs summarized in Table S1). These were, however, not significant after adjusting for multiple testing (LMM, FDR *p*_spectrum:time_ interaction > 0.99). We also tested the specific taxonomic changes for each antibiotic’s spectrum before and after treatment. Fourteen MGSs were nominally significant for the broad spectrum (Wilcoxon signed-rank test, unadjusted *p* < 0.05). Half of them were Clostridiales species together with three Bacteroides and *Faecalibacterium prausnitzii* and one *Eubacterium* enriched in post treatment (Table S2). For the narrow spectrum we found two species (*Clostridium phoceensis, Bacteroides uniformis*) significantly increased after treatment (Wilcoxon signed-rank test, unadjusted *p* < 0.05). However, none of these MGSs were significant after correction for multiple testing (FDR *p* > 0.21).

We finally analyzed whether there was taxon enrichment at pre- or post-treatment for both antibiotic treatments using TSEA. For the broad-spectrum treatment we found the Clostridiales order (RBC = 0.22, FDR *p* = 0.00011), Clostridia class (RBC = 0.22, FDR *p* = 0.00023) and Firmicutes phylum (RBC = 0.17, FDR *p* = 0.006) enriched in post-treatment while the Desulfovibrionales order (RBC = −0.80, FDR *p* = 0.014) and Deltaproteobacteria class (RBC = −0.80, FDR *p* = 0.010) were enriched in pre-treatment. For the narrow-spectrum treatment we found the Clostridiales order (RBC = 0.13, FDR *p* = 0.09), Clostridia class (RBC = 0.12, FDR *p* = 0.078) enriched in post-treatment while the Lactobacillales order (RBC = −0.37, FDR *p* = 0.09) and Bacilli class (RBC = −0.42, FDR *p* = 0.031) and Proteobacteria phylum (RBC = −0.34, FDR *p* = 0.069) were enriched in pre-treatment (Full analyses in supplementary_file_1.xlsx (broad) and supplementary_file_2.xlsx (narrow)).

### 3.4. General Increase of Resistome after Broad-Spectrum Antibiotic

**Figure 5:**
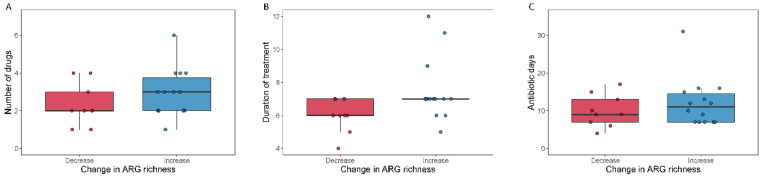
Number of antibiotics used (**A**), duration of antibiotic treatment (**B**) and total antibiotic days (**C**) relative to change in ARG richness. Boxplots show the number of antibiotics (**A**), the duration of treatment (**B**) and the total days on antibiotics (**C**) for patients who either decreased (“Decrease”) or increased (“Increase”) their ARG richness during broad-spectrum antibiotic treatment.

We detected 1093 (abundance > 0) antimicrobial resistance genes in the samples included in the analysis (*N* = 78). The abundance was summarized into 11 classes according to the type of antibiotics that they primarily confer resistance to (Table S3). Firstly, we investigated whether the antibiotic treatment changed the number of resistance genes in the human gut, by counting how many ARG genes were detected in each sample (ARG richness). Figure S4 shows the ARG richness for the pre- and post-treatment samples for each antibiotic spectrum treatment. We did not find significant differences between pre- and post-treatment for any of the two treatments (Wilcoxon signed-rank test; *p* > 0.17). For the broad-spectrum treatment there were no differences between the decreased and the increased ARG richness groups (>10 ARGs) with respect to number of drugs or selection pressure (Mann–Whitney U-test, *p*_number of drugs_ = 0.28, *p*_antibiotic days_ = 0.54), but we observed a significantly longer duration of treatment in the increased ARG richness group (Mann–Whitney U-test; *p*_duration of treatment_ = 0.026) (Figure 5). We next summed all the ARG abundances detected in each sample and tested whether the antibiotic treatment caused changes in the proportion of ARG genes (total ARG abundance Figure S4). We did not observe significant differences within the broad-spectrum (Wilcoxon signed-rank test; *p* = 0.53) nor within the narrow-spectrum treatment (Wilcoxon signed-rank test; *p* = 0.16).

We tested whether the resistome analyzed at the level of ARG classes was changing over time in a different manner for the two antibiotic treatments using LMM. We did not find significant differences in any of the 11 classes tested (LMM; FDR *p*_spectrum:time_ > 0.48). We did not find significant differences between the antibiotic spectrum treatments at pre- and at post-treatment (Wilcoxon signed-rank test; *p* > 0.10). However, we observed that most of the antibiotic class resistance genes were higher in abundance in post-treatment after broad-spectrum treatment (with the exception of Fluoroquinolones—Figure S5 and supplementary_file_3.xlsx), while most of the antibiotic classes’ resistance were enriched in pre-treatment after narrow-spectrum treatment (with the exception of Glycopeptides—Figure S5 and supplementary_file_3.xlsx).

### 3.5. Vancomycin Resistance Gene Richness Tend to Increase after Antibiotic Treatment

**Figure 6:**
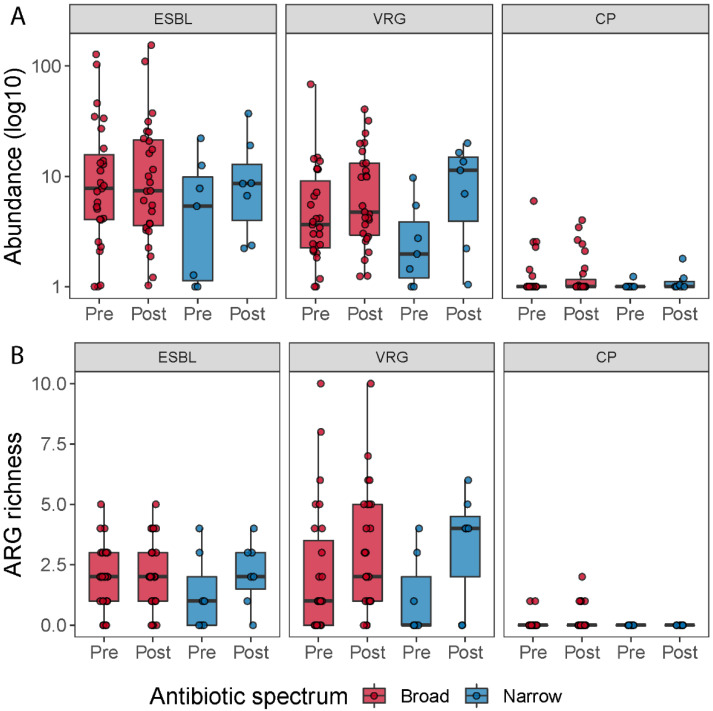
ARG richness for the ESBL, vancomycin resistance genes (VRG) and carbapenemase resistance gene groups (CP) (**A**). Boxplots show the resistance genes detected across the samples belonging to these three types of resistance genes by time and treatment. ESBL, vancomycin resistance and carbapenemase-producing resistance gene abundance for each treatment (**B**). Boxplots show the abundance of resistance genes (in log 10 scale) associated with ESBL, vancomycin resistance genes and carbapenemase resistance genes pre- and post-treatment, respectively.

To determine how many patients carried resistance genes belonging to extended-spectrum beta-lactamases (ESBL), vancomycin resistance genes (VRG) and carbapenemase-production (CP), we grouped all the beta-lactamases, except the ones that hydrolyze carbapenem and cephamycins into the ESBL group (in total 36 genes), all vancomycin resistance genes (in total 216 genes) and all carbapenem resistance genes into the CP group (in total 10 genes). We then counted how many patients carried at least one resistance gene belonging to one of these three groups at pre- or post-treatment; how many resistance genes assigned to these classes were detected (abundance > 0) in the samples; and we summed up the abundances of genes belonging to each of the three groups, to investigate the change in abundance of these groups during the antibiotic treatment (Figure 6). When we compared ESBL, VRG and CP richness and abundance between pre- and post-treatment for each antibiotic spectrum, we did not find any significant statistical difference (Table S4). However, when we pooled all samples belonging to pre- and post-treatment, irrespective of the treatment, and compared the two timepoints, we observed a tendency of a higher number of vancomycin resistance genes post-treatment (Wilcoxon signed-rank test; *p* = 0.063).

## 4. Discussion

In this study, we evaluated the effect of common antibiotics on the microbiome composition, diversity and resistome in patients admitted to NICU, in a cohort containing 34 cases and 5 controls, which were suitable for these analyses. This was a non-intervention study and patients were included from clinical settings investigating actual antibiotic treatment regimens routinely administered at NICU rather than not standardized or controlled regimens. Consequently, the study is biased towards the utilization of broad-spectrum antibiotics (broad *n* = 27; narrow *n* = 7), which may affect the power of our analyses. Additionally, in this study there was no recruitment or selection process; thus, the effect of the patient health condition improvement or worsening on the microbiome could not be controlled.

Our control cohort was small, but we observed compositional changes even in the control individuals. This suggests that all patients admitted to NICU are at risk of having their colonization resistance affected. This may be due to the limited bowl movement seen in these patients, the altered diet or due to use of other medical drugs, which can interfere with microorganisms in the gut. We need to include more participants in a new study to investigate this further and confirm the observation.

We observed a significant increase in diversity for patients in the narrow-spectrum group as well as a tendency for an increase in the Shannon index, which may indicate that the removal (killing) of some specific bacterial species allowed for other species to proliferate and occupy the emptier niche, maintaining what could be viewed as a well-balanced microbiota. Supporting this we observed a nominally significant increase of 15 microbes, mostly from the Bacteroides and Clostridium genera, in the narrow-spectrum treatment, which only maintained their levels in the broad-spectrum treatment. In contrast, we found no statistically significant differences in diversity (neither as richness nor as Shannon index) within the broad-spectrum antibiotic group, and generally the richness and Shannon index were more varying in this group. There was, however, a tendency among the patients with decreased richness and Shannon diversity to have had administered a higher number of drugs. It would have been expected that the broad-spectrum treatment had a larger effect on the intestinal composition compared to a narrower treatment. This was not the case and could reflect non-homogenous patient group characteristics (i.e., degree of illness, number of parallel treatments or number of medical drugs used at the same time) as there was no controlled standardized recruitment process. We had too few primary diagnoses recorded to compare between groups, yet for the recorded primary diagnoses we found no differences. Three patients in the increased group had a comorbidity noted regarding alcohol overconsumption, which is known to lower the diversity. One could speculate that stopping alcohol consumption during hospitalization could lead to an increase in diversity; however, this should be investigated in a new study.

For broad-spectrum patients, selection pressure (as antibiotic days) was significantly higher in patients with decreased richness and decreased Shannon diversity; these patients tended to have received a higher number of antibiotics or have been more days on antibiotics. A decrease in MGS was significantly correlated to the number of drugs administered and the selection pressure of the patient. Compositional differences between the pre- and post-treatment of samples in the narrow group were globally significant, indicating that the longer the narrow-spectrum antibiotic treatment, the higher the differences between the pre- and the post-treatment bacterial composition.

In this study we observed *Clostridiales* spp. increasing after treatment regardless of broad and narrow treatment. Opportunistic pathogens with antibiotic resistance potential from different clades or from *Clostridiales* (including *Clostridium difficile)* may proliferate during antibiotic treatment. Hospitalized patients treated with broad-spectrum or several antibiotics courses as part of their treatments are at higher risk of resistance bacteria colonization and hospital-acquired gastroenteritis. Consequently, the elimination of susceptible commensal species may allow for proliferation of resistant microbes, as has been previously described [3,5,13].

### 4.1. Impact of Antibiotic Treatment on Antimicrobial Resistance Genes

Overall, in this dataset there was an increase in the resistome in the broad-spectrum treatment, especially on subjects exposed to antibiotics for longer; but, we may not reach significance in most of the tests due to the high individual variation and the sample size (especially in the narrow group *N* = 7).

As most patients carried at least one ARG belonging to the ESBL or the vancomycin resistance gene groups, it is likely that many patients have been exposed to antibiotics before or have acquired bacteria carrying resistance genes. Our study therefore confirms that, in patients colonized with resistant microbes, antibiotics may increase the overall gene pool of resistance genes—but the level of increase in ARG richness depends on the class of ARG present and the antibiotics administered [7]. Thus, antibiotics of narrow as well as broad spectrum can select for resistance, proving the importance of a short-duration treatment [13,14]. This was further supported by our study, as the change in resistome follows largely the taxonomic compositional change, particularly among the broad-spectrum-treated patients. However, there are some discrepancies explained by other events, e.g., mobile elements carrying resistance genes or strain replacements. There was wide individual variation in the change in total ARG abundance across samples, again indicating the proliferation of species present in the gut prior to treatment.

In Denmark, a country of low prevalence of antimicrobial resistance [15], antibiotic naive patients rarely carry MDR bacteria [16]. Yet, antibiotic treatment may increase this risk of colonization as diversity and taxonomical composition is impacted. We did not identify a change in the antibiotic resistance gene prevalence in patients during treatment or admission, but this could be caused by the short duration of our study. It would be of great interest to perform a longitudinal investigation of antibiotic naive patients as well as patients with frequent hospital contact. Our study shows the importance of including several treatment groups as well as controls and illustrates that broad and narrow treatments affect the microbiome differently. In Denmark, we have seen an increase in the prevalence of vancomycin resistant *Enterococcus* mostly linked to hospital admission [15]—and when all patients are prone to colonization due to taxonomical changes, hospital infection control is critical. Therefore, in a clinical setting, it is relevant to investigate whether the prevalence of vancomycin resistance genes, ESBLs and carbapenemase resistance genes increase after antibiotic treatment only. Comparing across treatment in pre- and post-treatment, we observed that the vancomycin resistance genes richness is significantly higher in post-treatment. This group of van-genes includes not only the van-genes found in *Enterococcus faecium* but all van-genes; i.e., this difference does not represent growth of *E. faecium* alone rather the shift in taxonomy discussed above.

### 4.2. Study Limitations

The high levels of *Corynebacteriaceae* that we saw in few patients are likely due to the high levels of *Corynebacteriaceae* and *Corynebacterium* spp., which are usually found on human skin [17]. This could reflect the utilization of rectal swabs, as it has been seen that this sampling process is more prone to capture members of the skin microbiome compared to classic stool samples [18]. Oppositely, obtaining a normal fecal sample from this group of critically ill patients is often not possible as the bowel movement stops during intensive care treatment. Therefore, to ensure the sample collection at the same time across the study rectal swabs were the best choice.

The samples of this study were taken after 5–7 days of antibiotic treatment. It is possible that sampling further apart would have increased the possibility of distinguishing the long-term consequences of broad and narrow treatment on the resistome and microbiome. The skewed number of participants in the broad and narrow treatment groups may have affected the power of our analyses. In addition, a few samples exhibited fewer microbiome reads due to a high content of human reads (5 samples fell below 5 million microbial reads). However, the richness and Shannon index exhibited little correlation to sequencing depth (Kendall tau = 0.16 and 0.13, respectively). Although we cannot rule out that deeper sequencing of these samples may lead to improved resistance gene and species detection in these few samples, the overall conclusions drawn from the present study are not affected.

Finally, patients in the NICU are severely ill and are administered a lot of medication other than antibiotics. This lowers the ability to conclude on the effect of the antibiotics alone, but on the other hand underscores the importance of a study such as the current one in real-life settings.

## 5. Conclusions

Antibiotic naive, neurocritically ill patients exhibited differential effects in the taxonomic composition and resistome of their microbiomes. Patients who received narrow-spectrum treatment exhibited an increase in diversity. Selection pressure and duration of treatment was higher in patients with a decreased MGS diversity. The number of antibiotics administered to a patient and selection pressure were correlated to decreased MGS diversity and proved to be predictive of the effect on the microbiome independent of the antibiotic spectrum; i.e., a higher number of antibiotic days resulted in significantly decreased diversity. There was likewise a decreased richness and Shannon diversity in patients administered a higher number of drugs. Both findings support the purpose of antibiotic stewardship.

## Figures and Tables

**Table 1:** Overview of the applied antimicrobial treatments.

Antimicrobial Treatment	Number of Patients
Broad	
Cefuroxime, linezolide, meropenem	1
Piperacillin/Tazobactam	3
Cefuroxime, Erythromycin, Piperacillin/Tazobactam	3
Cefuroxime, Piperacillin/Tazobactam	1
Erythromycin, Piperacillin/Tazobactam, Metronidazole	1
Dicloxacillin, Piperacillin/Tazobactam	3
Ciprofloxacin, Piperacillin/Tazobactam	1
Erythromycin, Piperacillin/Tazobactam	3
Cefuroxime, Dicloxacillin, Erythromycin, Piperacillin/Tazobactam	1
Dicloxacillin, Piperacillin/Tazobactam, mero, clari	1
cipro, genta, Piperacillin/Tazobactam, vanco	1
Cefuroxime, Erythromycin, Meropenem, Piperacillin/Tazobactam	1
Erythromycin, Meropenem	1
Ampicillin, Piperacillin/Tazobactam	1
Dicloxacillin, Erythromycin, Meropenem, Piperacillin/Tazobactam	1
Ciprofloxacin, Pivmecillinam, Piperacillin/Tazobactam	1
Benzylpenicillin, erythromycin, Meropenem, Vancomycin	1
Erythromycin, Gentamycin, Piperacillin/Tazobactam	1
Narrow	
Cefuroxime, Erythromycin	1
Cefuroxime, Pivmecillinam, Vancomycin	1
Pivmecillinam	1
Ampicillin, Dicloxacillin, Cefuroxime	1
Benzylpenicillin	1
Erythromycin, Gentamicin, Meropenem, Piperacillin/Tazobactam, Vancomycin	1
Cefuroxime	2

**Table 2:** Correlation among the Bray–Curtis dissimilarities between the same-patient samples and antibiotic treatment characteristics (*n* = 34). Spearman correlation parameter (Rho) and correlation *p*-values are provided for each correlation.

Group	Number of Drugs	Duration of Treatment	Antibiotic Days
All subjects	0.03 (*p* = 0.86)	0.17 (*p* = 0.32)	0.20 (*p* = 0.24)
“Narrow” patients	0.78 (*p* = 0.038)	0.32 (*p* = 0.47)	0.77 (*p* = 0.039)
“Broad” patients	−0.08 (*p* = 0.68)	−0.22 (*p* = 0.25)	0.037 (*p* = 0.85)
“Broad” Decreased diversity	0.085 (*p* = 0.79)	0.20 (*p* = 0.54)	0.14 (*p* = 0.66)
“Broad” Increased diversity	0.058 (*p* = 0.83)	−0.070 (*p* = 0.82)	0.027 (*p* = 0.92)

## Data Availability

The raw data behind this study is deposited in ENA under Accession PRJEB48897.

## Supplementary Materials

The following are available online at https://www.mdpi.com/article/10.3390/microorganisms9122542/s1, Figure S1: Barplots summarizing read quality and read mapping, Figure S2: Duration of treatment, number of drugs and antibiotic days across samples by antibiotic spectrum group, Figure S3: MGSs that changed differently between antibiotic spectrum over time, Figure S4: ARG richness and total abundance, Figure S5: Antibiotic resistance gene classes abundance for each treatment, Table S1: Statistics related to the comparison between antibiotic spectrum groups over time, Table S2: specific taxonomic changes for each antibiotic spectrum before and after treatment, Table S3: Antibiotic resistance genes detected from each antibiotic class in the patient samples, Table S4: The *p*-values for the ESBL, VRG and CP richness and abundance comparison between pre- and post-treatment for broad and narrow-spectrum groups, supplementary_file_1: taxon enrichment at pre- or post-treatment for broad antibiotic treatments using TSEA, supplementary_file_2: taxon enrichment at pre- or post-treatment for narrow antibiotic treatments using TSEA, supplementary_file_3: resistome analyzed at the level of ARG classes over time using LMM.
