# Supplementary material for: Microbiome Compositions and Resistome Levels after Antibiotic Treatment of Critically Ill Patients: An Observational Cohort Study
Source: Microorganisms. 2021 Dec 9;9(12):2542. doi: 10.3390/microorganisms9122542 (PMC8703874; doi:10.3390/microorganisms9122542)
Supplement: Supplementary file 1 [file microorganisms-09-02542-s001.zip › Supplementary Tables_09122021.pdf]

Supplementary Tables

Table S1. Statistics related to the comparison between antibiotic spectrum groups over time. Unadjusted *p*-values from the LMM (p), and fold changes (FC; calculated as mean of log2 abundance ratios) are shown for the 15 MGS that exhibited a significant unadjusted p-value in the comparison.

MGS	Species	p_{spec}	P_t	$p_{spec:t}$	FC "B"	FC "N"
MGS.hg0886	*Bacteroides sp.*	0.06	0.11	0.00	-0.04	0.78
MGS.hg0921	*Bacteroides sp.*	0.48	0.85	0.01	0.54	-0.16
MGS.hg0976	*Clostridiales sp.*	0.10	0.23	0.01	0.66	-0.54
MGS.hg0716	*Clostridiales sp.*	0.87	0.11	0.01	0.56	0.36
MGS.hg0334	*Bacteroides cellulosilyticus*	0.86	0.18	0.02	-1.17	1.18
MGS.hg1138	*Clostridiales sp.*	0.73	0.73	0.02	-0.33	0.61
MGS.hg0169	*Bacteroides intestinalis*	0.04	0.13	0.02	-0.80	2.52
MGS.hg0559	*Clostridiales sp.*	0.24	0.50	0.02	-0.72	3.42
MGS.hg0772	*Clostridiales sp.*	0.30	0.55	0.02	0.16	-1.55
MGS.hg0930	*Clostridiales sp.*	0.84	0.27	0.03	-0.47	1.43
MGS.hg0170	*Clostridiales sp.*	0.18	0.29	0.03	-0.53	1.47
MGS.hg1047	*Clostridiales sp.*	0.82	0.14	0.04	0.46	0.58
MGS.hg0004	*Blautia wexlerae*	0.25	0.12	0.04	1.96	5.18
MGS.hg0593	*Clostridiales sp.*	0.58	0.62	0.04	0.47	2.06
MGS.ref1184	*Actinomyces oris*	0.40	0.06	0.05	0.26	1.71

Table S2: specific taxonomic changes for each antibiotic spectrum before and after treatment.

MGS	Species	*p*-value	FDR	Higher in	FC
Broad					
MGS.hg0207	*[Clostridium] leptum*	0.0004	0.21	Post	2.97
MGS.hg0365	*Clostridiales sp.*	0.001	0.38	Post	3.03
MGS.hg0002	*Bacteroides uniformis*	0.004	0.79	Post	2.08
MGS.hg0643	*Clostridiales sp.*	0.017	1	Post	1.96
MGS.hg0323	*Bacteroides uniformis*	0.026	1	Post	1.59
MGS.hg0700	*Clostridiales sp.*	0.026	1	Post	2.03
MGS.hg0018	*Faecalibacterium prausnitzii*	0.027	1	Post	2.64
MGS.hg0695	*Clostridia sp.*	0.035	1	Post	1.56
MGS.hg0126	*Eubacterium sp.*	0.038	1	Post	3.82
MGS.hg0013	*Ruminococcus bromii*	0.043	1	Pre	-1.27
MGS.hg0145	*Faecalibacterium prausnitzii*	0.044	1	Post	1.97
MGS.hg0006	*Bacteroides ovatus*	0.048	1	Post	1.52
MGS.hg0309	*Clostridiales sp.*	0.050	1	Post	1.67
MGS.hg0839	*Clostridiales sp.*	0.050	1	Post	3.04
Narrow					
MGS.hg1043	*Clostridium phoceensis*	0.015	0.98	Post	5.31
MGS.hg0002	*Bacteroides uniformis*	0.031	0.98	Post	4.54

Table S3. Antibiotic resistance genes detected from each antibiotic class in the patient samples (n=78)

Antibiotic class	Number of genes detected for each antibiotic class	Number of genes annotated in the 22M catalog
Aminoglycoside	55	85
Beta-lactam	35	59
Chloramphenicol	88	143
Fluoroquinolone	9	12
Glycopeptide	147	216
Lincosamide	34	50
Macrolide	24	40
Multidrug efflux pumps	503	886
Spectinomycin	55	85
Streptogramin	20	30
Tetracycline	120	173
Other	545	1008

Table S4. The p-values for the ESBL, VRG and CP richness and abundance comparison between pre- and post-treatment for broad and narrow-spectrum groups. These p-values correspond to Wilcoxon signed-rank tests run for each of the resistance groups within each antibiotic spectrum group. "NA" is shown when a *p*-value could not be calculated (for example CP richness is 0 in both groups).

Microbial feature	Antibiotic spectrum	ESBL	VRG	CP
ARG abundance	Broad	0.90	0.50	1
	Narrow	0.47	0.22	0.79
ARG richness	Broad	0.62	0.20	0.07
	Narrow	0.29	0.10	NA
