# Supplementary figures and images for: Microbiome Compositions and Resistome Levels after Antibiotic Treatment of Critically Ill Patients: An Observational Cohort Study
Source: Microorganisms. 2021 Dec 9;9(12):2542. doi: 10.3390/microorganisms9122542 (PMC8703874; doi:10.3390/microorganisms9122542)

QC and read mapping summary

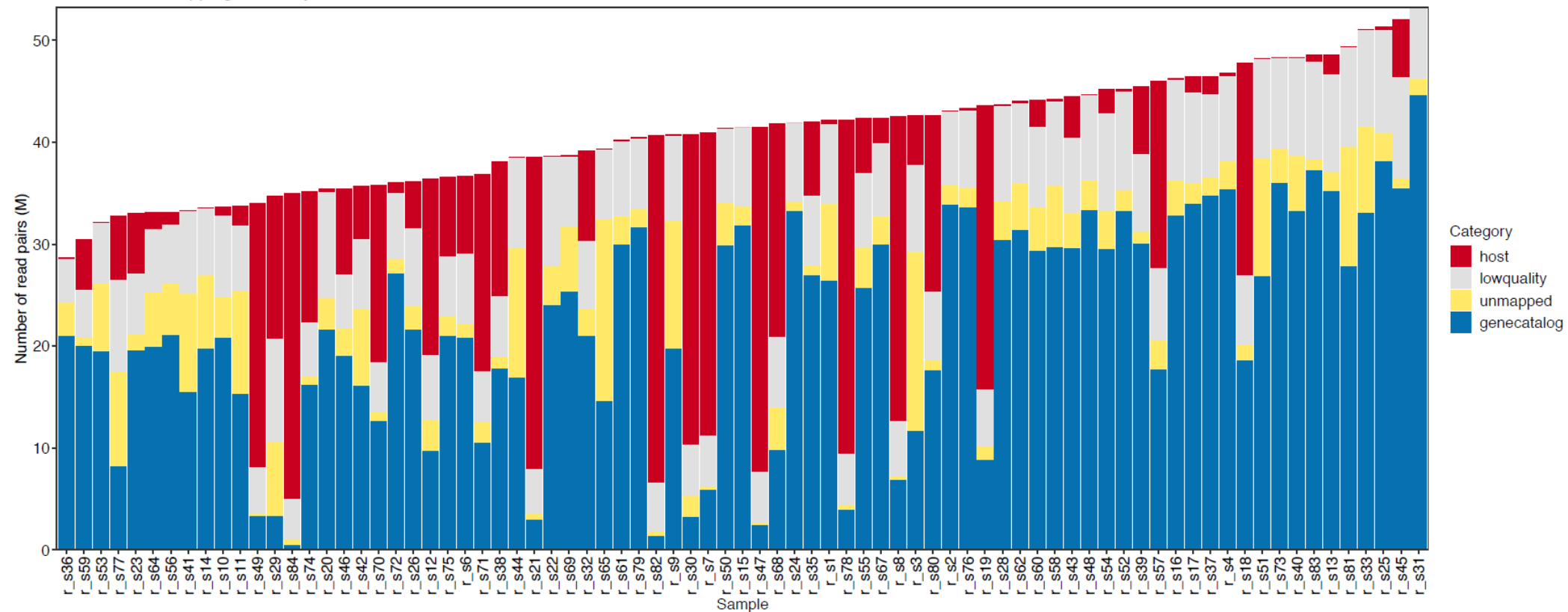

Supplement: Supplementary file 1 [file microorganisms-09-02542-s001.zip › Figure S1.pdf]

Duration of treatment

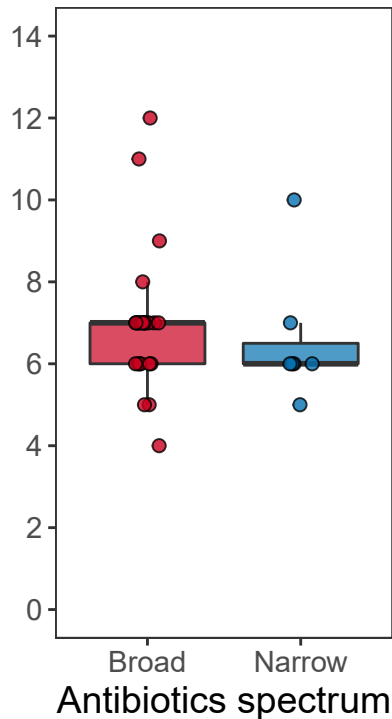

Number of drugs

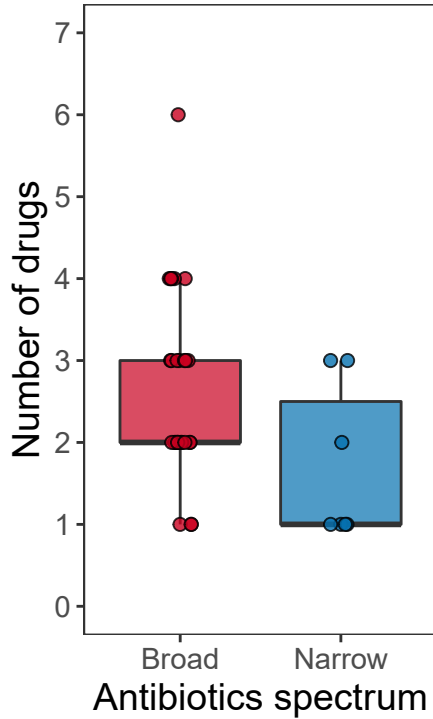

Antibiotic days

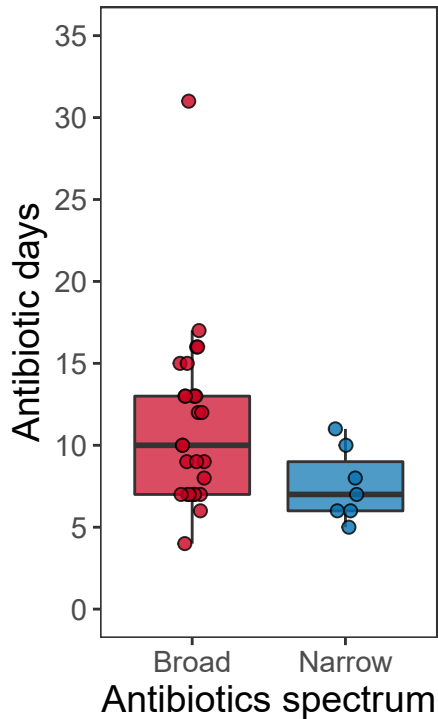

Supplement: Supplementary file 1 [file microorganisms-09-02542-s001.zip › figure S2.pdf]

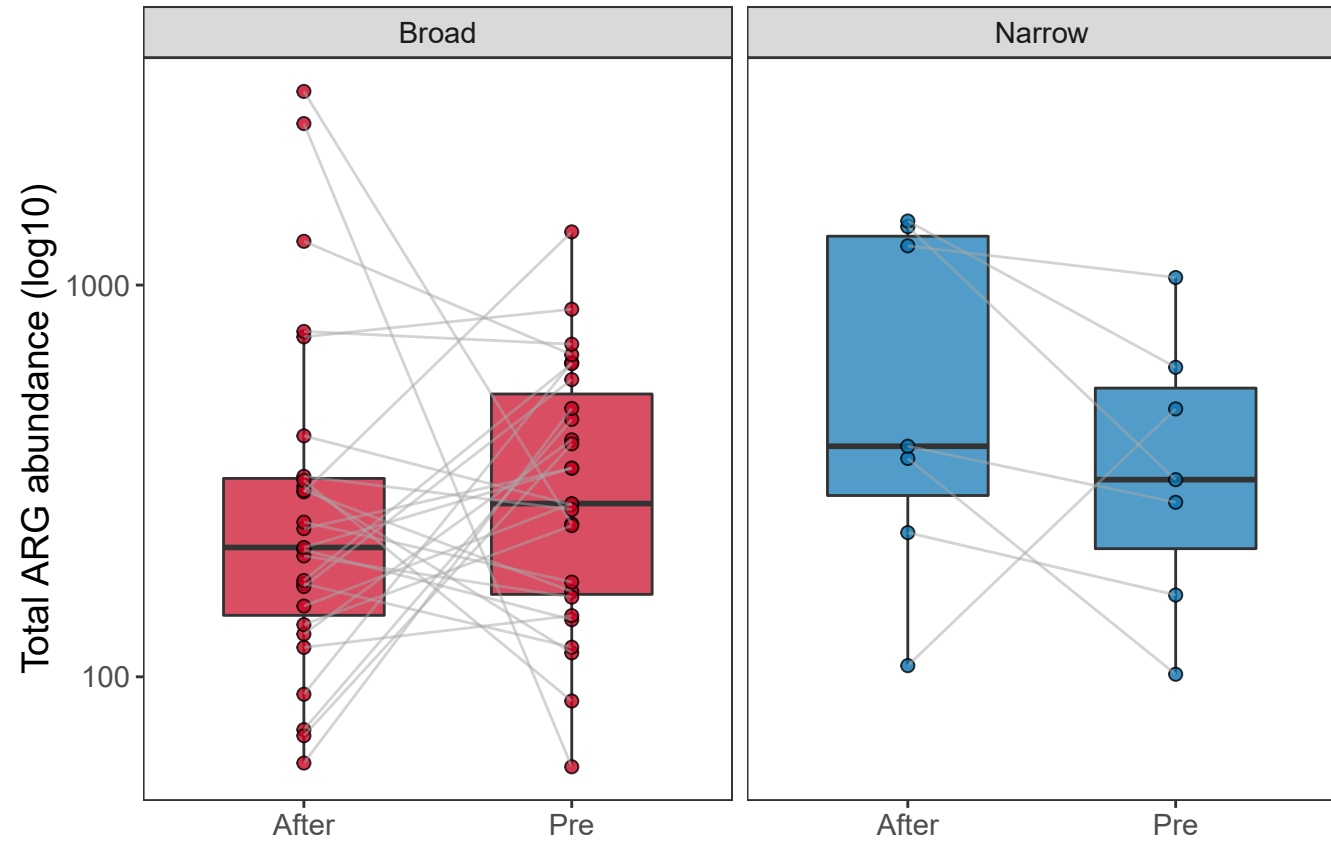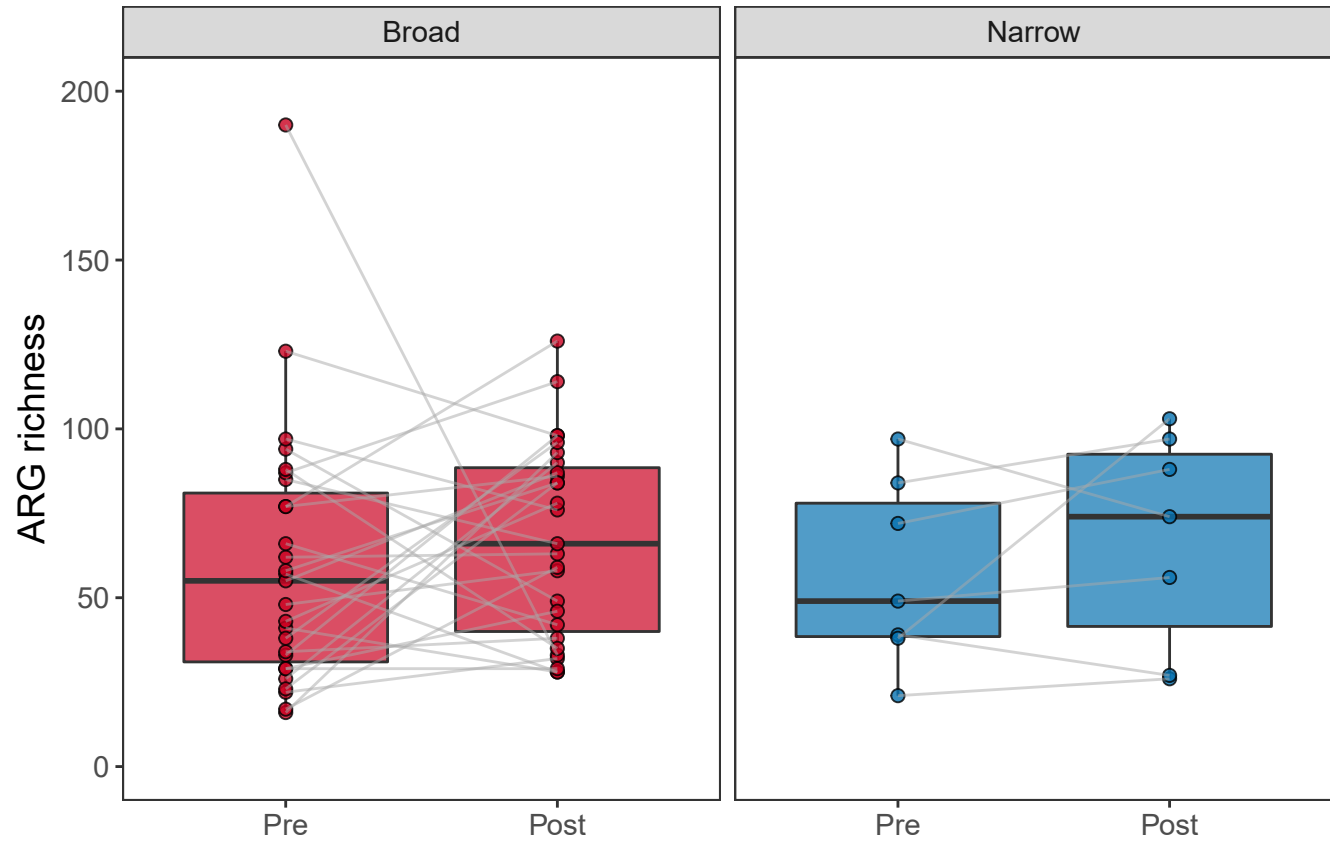

Supplement: Supplementary file 1 [file microorganisms-09-02542-s001.zip › Figure S4.pdf]

# MGS changing differently between treatments over time

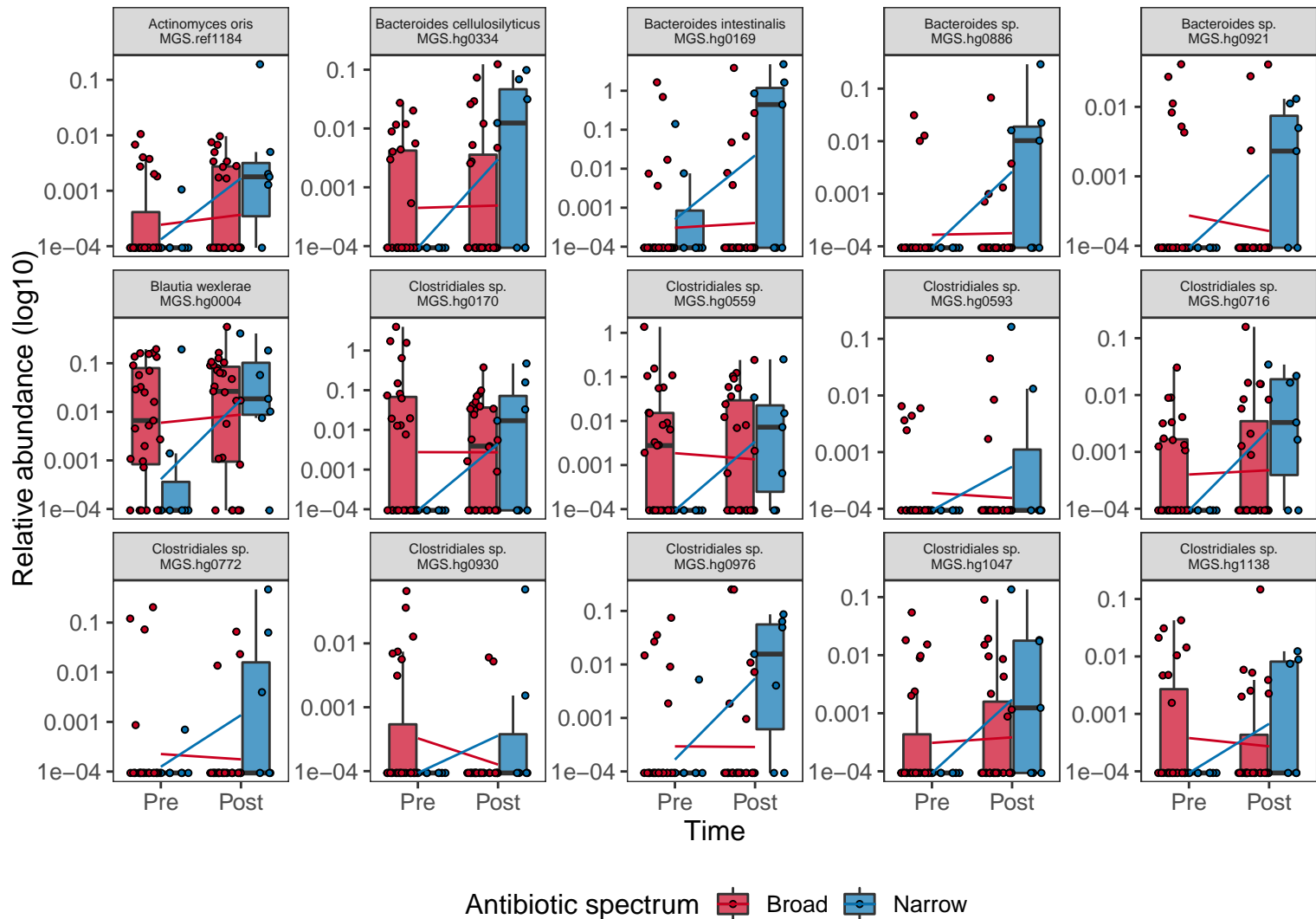

Supplement: Supplementary file 1 [file microorganisms-09-02542-s001.zip › FigureS3_LMM_significants.pdf]

Abundance (log10)

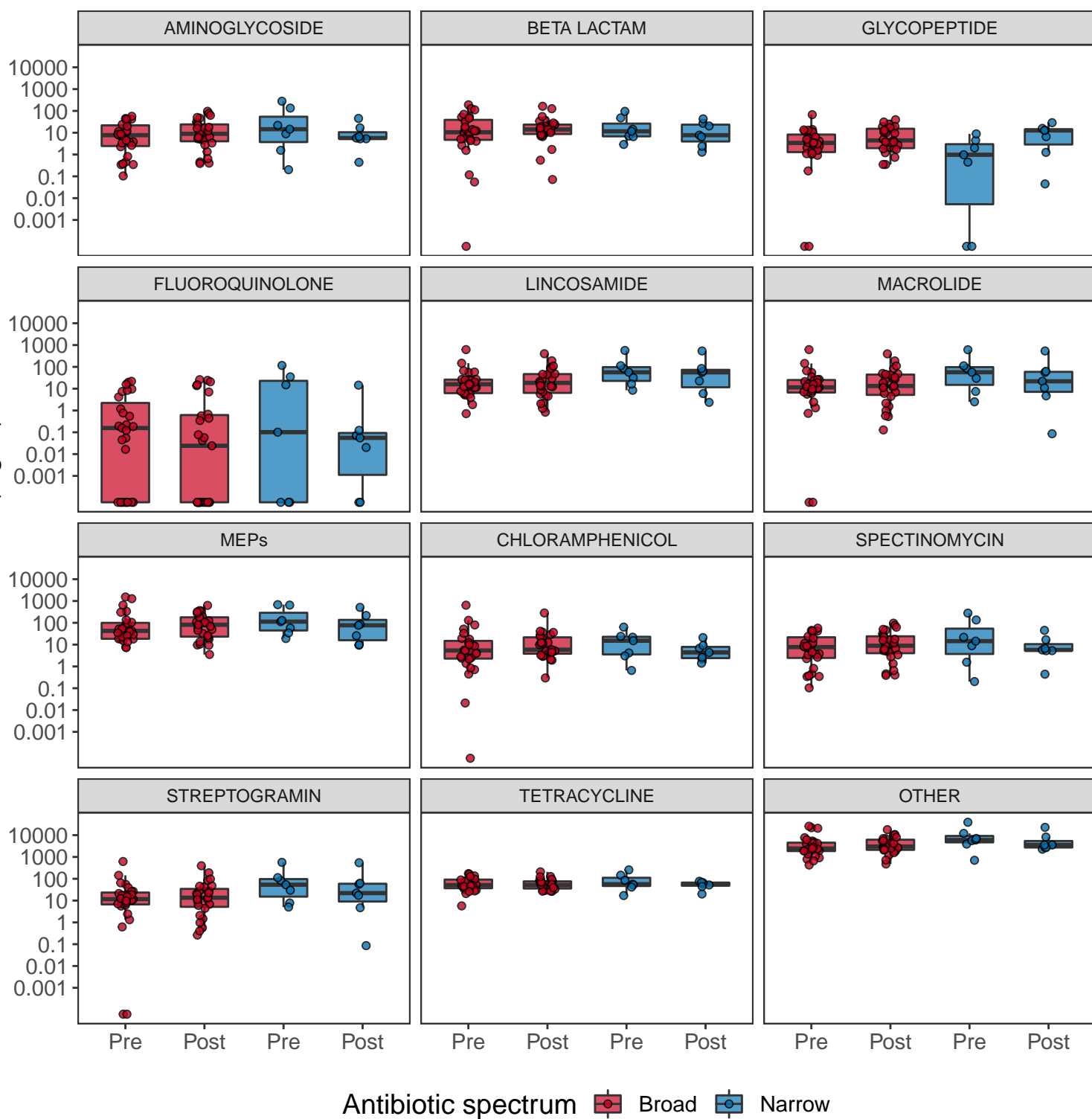

Supplement: Supplementary file 1 [file microorganisms-09-02542-s001.zip › FigureS5_ABX_class.pdf]
